# Mitogenome of *Medicago lupulina* L. Cultivar-Population VIK32, Line MlS-1: Dynamic Structural Organization and Foreign Sequences

**DOI:** 10.3390/ijms262411830

**Published:** 2025-12-07

**Authors:** Maria E. Vladimirova, Marina L. Roumiantseva, Alla S. Saksaganskaia, Alexandra P. Kozlova, Victoria S. Muntyan, Sergey P. Gaponov, Andrey P. Yurkov, Vladimir A. Zhukov, Mikhail P. Grudinin

**Affiliations:** 1 Federal State Budget Scientific Institution All-Russia Research Institute for Agricultural Microbiology (FSBSI ARRIAM), 196608 Saint Petersburg, Russia; mariiacherkasova@arriam.ru (M.E.V.); allasaksaganskaya@arriam.ru (A.S.S.); a.kozlova@arriam.ru (A.P.K.); vucovar@arriam.ru (V.S.M.); sp.gaponov@arriam.ru (S.P.G.); ap.yurkov@arriam.ru (A.P.Y.); vzhukov@arriam.ru (V.A.Z.); 2 Novikov Labs, 420033 Kazan, Russia; 3 Smorodintsev Research Institute of Influenza, Ministry of Health of the Russian Federation, 197376 Saint Petersburg, Russia; mikhail.grudinin@influenza.spb.ru

**Keywords:** plant mitochondrial genome, *Medicago lupulina*, MlS-1 line, VIK32 cultivar-population, *Medicago* spp., organellar phylogenomics, endosymbiosis, phylogenetic analysis, chloroplast DNA, horizontal gene transfer, intraspecific polymorphism

## Abstract

This study presents the complete assembly and analysis of the mitochondrial genome (mitogenome) of *Medicago lupulina* L. var. *vulgaris* Koch, cultivar-population VIK32, line MlS-1, which forms an effective symbiosis not only with arbuscular mycorrhiza but also with the root nodule bacteria *Sinorhizobium meliloti*. The assembly, generated using a hybrid sequencing approach, revealed sequences of putative horizontal origin. These include a highly conserved open reading frame (ORF), *orf279*, encoding a protein structurally homologous to maturase K, yet bearing remote similarity to bacterial reverse transcriptases and CRISPR-associated proteins. We also identified sequences homologous to mitovirus RNA-dependent RNA polymerases and a fragment of the chloroplast 23S ribosomal RNA (rRNA), suggesting historical gene transfers from viruses and plastids. This work establishes a foundation for investigating the role of mitochondrial genome variation in key plant’s phenotypic traits, such as the enhanced responsiveness to arbuscular mycorrhiza observed in this agronomically valuable line.

## 1. Introduction

The genomes of mitochondria and chloroplasts serve as unique models for addressing fundamental questions in evolutionary genomics, particularly those concerning endosymbiosis. Advances in next-generation sequencing, especially long-read technologies such as Nanopore, have provided unprecedented opportunities for plant organelle research. The study of these organelles of bacterial origin is opening new frontiers in understanding plant evolution, adaptation, and functional traits [1,2].

As energy-producing organelles of eukaryotic cells, mitochondria originated from the integration of protomitochondria, derived from α-proteobacteria, into a host cell [3,4]. This pivotal event in eukaryotic evolution triggered a radical metabolic reorganization, leading to a reliance on aerobic pathways for energy production and biosynthetic precursor synthesis [5].

In plants, mitochondria are characterized by extremely large genomes, in contrast to the conservative and compact mitochondrial genomes (mitogenomes) of animals. Plant mitogenomes exhibit structural plasticity, reflected in significant size variation—ranging from 66 thousand base pairs (kilobase, kb) in *Viscum scurruloideum* to a record 11.7 million base pairs (Mb) in Siberian larch, *Larix sibirica* [6,7]. It has been suggested that this “size paradox” is due to a large amount of non-coding sequences, repeats, and mobile genetic elements, although their collective contribution does not exceed 11% of the genome size [7]. The mitogenomes of angiosperms are typically represented by a single circular chromosome, often referred to as the “master chromosome” [8]. However, recent research indicates that plant DNA in vivo exists as a dynamic, recombining pool of circular and non-circular (linear, branched) forms [2,8,9]. Despite considerable size variation, plant mitogenomes encode a small, conserved set of genes essential for respiration, translation, and other core functions—typically including subunits of oxidative phosphorylation complexes, ribosomal proteins, and transfer RNAs (tRNAs) [10,11,12,13]. For example, the larch mitogenome contains 40 protein-coding genes, 34 tRNAs, and 3 ribosomal RNAs (rRNAs), a composition representative of plant mitochondria [7]. However, mitogenome characteristics can vary significantly across different families. For instance, the mitogenome of safflower (*Carthamus tinctorius* L. 1753, *Asteraceae*)—a valuable cash crop with both edible and medicinal properties—contains 55 unique genes, including 34 protein-coding genes (PCGs), 3 rRNA genes, and 18 tRNA genes [2]. It is suggested that plant mitochondrial genomes are characterized by high recombinational activity, driven by numerous repeats. Against a background of low mutation rates and limited coding capacity, this activity leads to a punctuated mode of evolution. This evolutionary process occurs in dramatic bursts, accompanied by large-scale rearrangements and the loss and gain of DNA sequences [14,15]. The slow nucleotide substitution rate and conserved gene content make mitogenomes particularly valuable for phylogenetic studies.

Functional integration of mitochondria has occurred mainly with the nucleus, although growing evidence also indicates historical genetic exchange between mitochondria and plastids. However, comparative studies of mitogenomes and chloroplast genomes are often conducted on non-overlapping sets of individuals—different varieties, ecotypes, or genetic lines of the same species. This lack of parallel data complicates evolutionary interpretation and underscores the need for coordinated organelle genome sequencing.

The genus *Medicago* (*Fabaceae*) is of major agricultural and ecological importance, comprising species such as the forage crop alfalfa (*M. sativa*) and the model legume *M. truncatula*. To date, the number of sequenced *Medicago* spp. genomes with varying levels of assembly has reached 37 in GenBank (as of 29 October 2025). However, only nine complete mitochondrial assemblies are available, including that of *M. truncatula* [16,17], compared to 19 complete chloroplast genomes [18]. This pronounced data imbalance limits robust comparative and phylogenetic analyses, hindering a comprehensive understanding of organellar evolution in the genus.

*Medicago lupulina* (black medick) is a widely prevalent species valued as a pasture crop in cold humid climates. It also occurs across diverse eco-geographical regions and can grow in degraded soils [19,20,21]. The cultivar-population *M. lupulina* VIK32, derived from a Kyrgyzstan (Central Asia) ecotype, effectively nodulates with *Sinorhizobium meliloti* [22] and has been used to breed lines showing high responsiveness to arbuscular mycorrhizal fungi.

Here, we present the assembly and analysis of the mitogenome of the selected line MlS-1, derived from VIK32. This line exhibits high responsiveness to *Rhizophagus irregularis* and, in the absence of the symbiont, shows dwarfism under phosphorus deficiency [23]. We previously reported the plastid genome of MlS-1 [18]. Integration of the new mitogenomic data revealed previously undocumented intraspecific polymorphism in *M. lupulina* and elucidated the distinctive structural features of its mitochondrial DNA. These findings deepen our understanding of the diversity and evolution of organelles of bacterial origin in the genus *Medicago*.

## 2. Results

**Figure 1:**
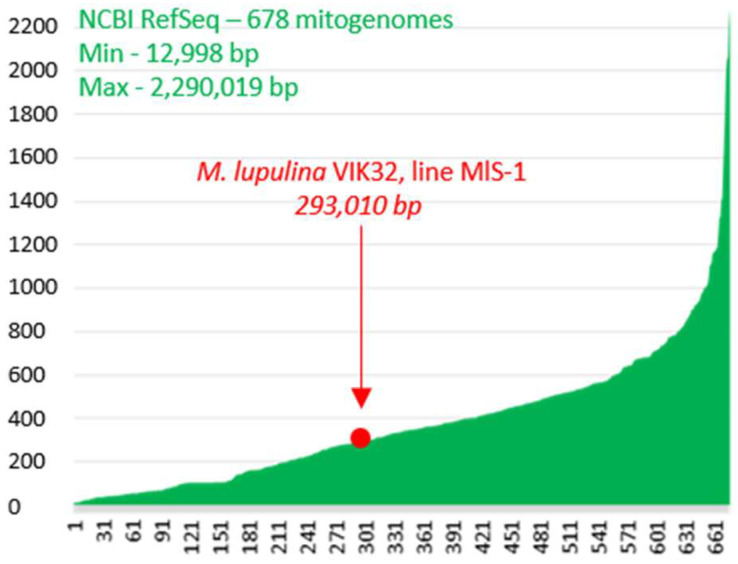
Plants mitogenomes. On the x-axis—678 plants mitogenomes from GenBank (RefSeq 30 October 2025), sorted by lengths ascending; on the y-axis—mitogenomes lengths (kb); Min—the minimum-size mitogenome (*Polytomella capuana*); Max—the maximum-size mitogenome (*Selenicereus monacanthus*). The *Larix sibirica* mitogenome with a length of 11.7 Mb is not shown in the figure.

Mitochondrial genome (hereafter mitogenome or mtDNA) sequence of the *M. lupulina* L. var. *vulgaris* Koch, cultivar-population VIK32, line MlS-1 (hereafter VIK32, line MlS-1) was obtained and deposited in GenBank. mtDNA of VIK32, line MlS-1 is a 293,010 base pairs (bp) long double-stranded circular DNA with a guanine-cytosine (GC) content of 45.34% (Figure 1). The mtDNA of line MlS-1 was found to be 1113 bp shorter than the mtDNA of wild-growing *M. lupulina* OY283147.1 (length 294,123 bp; GC content 45.4%) and was 164 bp larger than the mtDNA of wild-growing *M. lupulina* PV916040.1 (length 292,846 bp; GC content 45.4%). The pairwise similarity level of all 3 indicated *M. lupulina* mitogenomes was high (Cover 100%, Identity 99.59–99.87%) (Appendix B: Table A2). All studied *M. lupulina* mitogenomes (VIK32, OY283147.1 and PV916040.1) was over 20 kb larger, than the mtDNA of *Medicago truncatula* NC_029641.1 (271,618 bp; GC content 45.4%) long-established model for the study of legume biology according to [16,17].

### 2.1. Annotation, Structure and Order of Genes in mtDNA

#### 2.1.1. Annotation of mtDNA of VIK32, Line MlS-1

**Figure 2:**
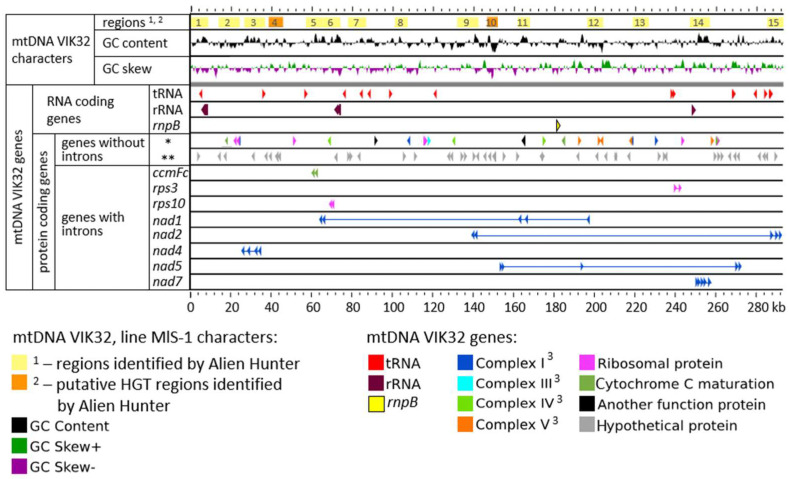
The mitogenome of *Medicago lupulina* VIK32, line MlS-1. * genes encoding proteins with predicted functions; ** genes encoding hypothetical proteins. ^3^—Complex I—NADH dehydrogenase; Complex III—cytochrome c reductase/cytochrome bc1 complex; Complex IV—cytochrome c oxidase; Complex V—ATP synthase.

mtDNA of VIK32, line MlS-1 contained: (i) 21 open reading frames (ORFs) encoded various RNAs, (ii) 33 protein-coding ORFs with predicted function and (iii) 45 ORFs coding hypothetical protein products (Figure 2).

(i). Annotation of RNA-Encoding Genes

The ORFs encoding RNA molecules were represented by four genes for rRNAs, 16 genes for tRNAs, and one gene for the RNA component of RNase P (Figure 2).

**Figure 3:**
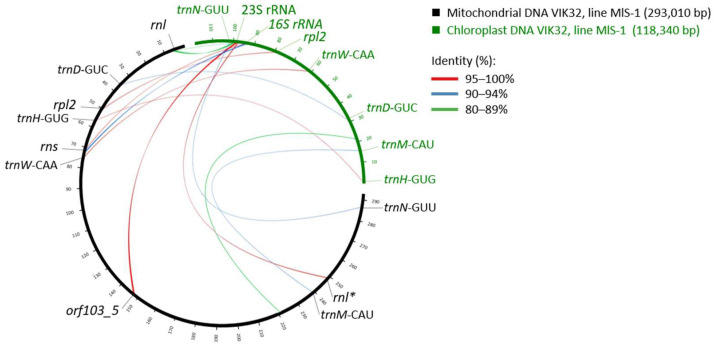
Schematic representation of syntenic regions between the VIK32, line MlS-1 mitochondrial and chloroplast genomes. Homologous regions are indicated. The asterisk (*) marks a gene fragment (described in the main text). For detailed nucleotide alignment data, see Table S1.

Of the 16 tRNA genes, 11 were found exclusively in the mitochondria (tRNA-K(UUU), -C(GCA), -Y(GUA), -F(GAA), -P(UGG), -E(UUC), -fM(CAU), -I(CAU), -Q(UUG), -G(GCC), -E(CUG)). The other five tRNA genes (tRNA-N(GUU), -M(CAU), -D(GUC), -H(GUG), -W(CCA)) were also identified in the chloroplast genome (Figure 3).

A sequence comparison of the mitochondrial and chloroplast tRNA genes revealed 100% identity for tRNA-W(CCA). For the remaining four common tRNAs, the sequence identity ranged from 93.15% to 98.65%.

Furthermore, two ORFs annotated as *rnl* (coordinates 248,447–248,926 and 5265–8440) exhibited similarity to different fragments of the chloroplast 23S rRNA gene. The sequence coverage was 38% and 34%, with identity levels of 80.5% and 100%, respectively. Another ORF, annotated as *rns* (coordinates 72,061–74,074), showed similarity to fragments of the chloroplast 16S rRNA gene (Cover 57%, Identity 90.2%; Figure 3).

Similarity with bacterial genes was identified for six tRNA-encoding ORFs and one rRNA-encoding ORF using BLASTn (BLAST+ 2.17.0) with default alignment parameters.

The ORFs encoding tRNA-N and tRNA-W, as well as one *rnl* gene fragment (coordinates 248,447–248,926), showed similarity to corresponding genes from various cyanobacteria (Cover 91–99%, Identity 89.6–97.3%). In contrast, a second *rnl* gene (coordinates 5265–8440) exhibited similarity to the corresponding gene in *Rhodospirillum rubrum* (Cover 36%, Identity 78.9%), a bacterium belonging to the *Rhodospirillaceae* family, which is the same family as the presumed mitochondrial ancestor. Furthermore, this particular *rnl* gene and the ORF encoding tRNA-D showed similarity to genes from *Candidatus* Liberibacter asiaticus (Cover 66% and 88%, Identity 80.1% and 98.6%, respectively), the causative agent of Huanglongbing (also known as citrus greening disease).

The ORF encoding *rns* (coordinates: 72,061–74,074) displayed similarity only to a 16S rRNA gene from an uncultured bacterium, while the *rrn5* gene (coordinates: 71,775–71,886) showed no significant similarity to any bacterial sequences.

Finally, ORFs encoding tRNA-F, -H, -Q, and -I demonstrated similarity to corresponding genes from *Candidatus* Zinderia (Cover 92%, Identity 91.2%), an uncultured bacterium clone from pesticide-contaminated soil (Cover 100%, Identity 96%; BioSample: SAMN03437033), *Mesorhizobium* spp. (Cover 74%, Identity 92.4%), and an uncultured bacterium from the Middle Tianshan Mountain (Cover 100%, Identity 100%; BioProject: PRJEB52793), respectively.

(ii). Annotation of Protein-Coding Genes

Among the 33 ORFs encoding proteins with predicted functions were: nine encoding NADH dehydrogenase subunits, one encoding cytochrome b, three encoding cytochrome C oxidase subunits, five encoding ATP synthase subunits, four responsible for cytochrome C maturation, nine encoding ribosomal proteins, and two with other functions (*matR* and *mttB*; Table 1, Figure 2). Among these, the *cox2*, *rps3*, *nad1*, *nad5*, and *rpl2* genes were characterized with potential mutations due to single nucleotide polymorphisms (SNPs). The *rpl2* sequence from the VIK32, line MlS-1 mtDNA showed identity with exon 2 of the chloroplast gene encoding ribosomal protein L2. For example, it showed identity with an exon of the line MlS-1 chloroplast gene *rplB* (OR674883.1; coordinates 78,587–79,084; Cover 69%, Identity 98.3%) (Figure 3), as well as with exon 2 of the chloroplast gene *rpl2* from *M. lupulina* (OM681370.1; Cover 72%, Identity 98.4%) and *M. truncatula* cultivar Jemalong A17 (MT965675.1; Cover 72%, Identity 97.5%). This sequence also showed high similarity to sequences from the chloroplast genomes of phylogenetically distant plants from the genera *Trigonella*, *Melilotus*, *Lathyrus*, and *Pisum* (Cover 97%, Identity 95.6–96.2%).

(iii). ORFs Encoding Hypothetical Proteins

The 45 protein-coding genes were characterized as genes encoding hypothetical proteins (Figure 2). Analysis of the amino acid sequences of hypothetical proteins revealed that two of them (*orf435*: 133,516–134,823 and *orf183*: 135,572–136,123) exhibited similarity to the mediator of RNA polymerase II transcription subunit 33A-like, for example, from *Fabaceae* family plant *Neltuma alba* (formerly as: *Prosopis alba*) according to BLASTp (BLAST+ 2.17.0) (Cover 97% and 84%; Identity 87.26% and 92.21%, respectively) and subunit 33A/B from *Arabidopsis* according InterProScan v5.76-107.0. Amino acid 3D-structure of this hypothetical proteins (*orf435* and *orf183* products) also demonstrate similarity with RNA polymerase II transcription subunit 33A-like from *Glycine max* (Seq Identity 70.30%, GMQE 0.72) and *Gossypium lobatum* (Seq Identity 76.73% GMQE 0.75), correspondingly, according to bioinformatics 3D-structure analysis using SWISS-MODEL Workspace/GMQE tool. So, this ORFs *orf435* and *orf183* were characterized as ORFs encoding RNA polymerase II transcription subunit 33A-like proteins.

A nucleotide sequence of *orf103*_*5* (*orf103*_*5*: 147,584–147,895) was identified that exhibited a high degree of similarity to a fragment of the 23S rRNA gene from the chloroplast genome of *Medicago* species (Cover 99–100%, Identity 97.44–100%). This included the reference plant *M. truncatula* (Cover 100%, Identity 98.08%) and *M. lupulina* VIK32, MlS-1 itself (Cover 100%, Identity 100%) (Figure 3). Significant similarity was also observed with chloroplast 23S rRNA genes from phylogenetically distant genera, including *Vicia*, *Astragalus*, *Trifolium*, *Lens*, *Lathyrus*, and *Pisum* (Cover 99–100%, Identity 97.76–99.68%).

Three other hypothetical proteins (*orf603*: 41,506–43,317, *orf111_3*: 77,973–78,308, and *orf115*: 266,237–266,584) showed similarity to the RNA-dependent RNA polymerase (RdRp) of mitovirus *Mitoviridae* sp., according to BLASTp (BLAST+ 2.17.0) (Cover 91%, 29%, and 64%; Identity 43.3%, 62.5%, and 30.4%, respectively). These are the mitochondria-replicating “naked RNA viruses” with genomes encoding only the replicase RNA-dependent RNA polymerase (RdRp) and prevalent across fungi, plants, and invertebrates [24]. This sequences (*orf603*, *orf111_3* and *orf115*) also demonstrate similarity with plants mitochochondrial proteins characterized as mitovirus RNA-dependent RNA polymerase, for example, *orf603* product was similar to *Shorea laevis* protein (GLT62820.1: Cover 73%, Identity 57.47%), *orf111_3* product was similar to *Lathyrus sativus* protein (CAK8563079.1: Cover 100%, Identity 97.30%) and *orf115* product was similar to walking iris protein (WDD62795.1: Cover 86%, Identity 34.95%). *orf603* product also exhibited similarity with different mitoviruses RNA-dependent RNA polymerase according to InterProScan v5.76-107.0. 3D-structure of *orf603* product was equally similar to RNA-directed RNA polymerase of viral and host proteins (Seq Identity 14.35%, GMQE 0.18, QMEANDisCo Global: 0.45 ± 0.05) and Partial Cas6-RT-Cas1–Cas2 complex (Seq Identity 14.21%, GMQE 0.12, QMEANDisCo Global: 0.34 ± 0.06). Products of *orf111_3* and *orf115* were similar to uncharacterized protein of different organisms or demonstrate a low similarity with proteins (Seq Identity < 35%, GMQE < 0.10). So, *orf603* was characterized as ORFs encoding potential mitovirus RNA-dependent RNA polymerase whereas *orf111_3* and *orf115* were characterized as ORFs encoding hypothetical proteins.

We identified a conserved 840 bp sequence (*orf279*: 128,472–129,311) that shares 100% identity (Cover 100%) with an unannotated region in the *Medicago lupulina* mitogenome (PV916040.1) and 99.88% similarity (Cover 100%) with a homologous region in the *M. sativa* mitogenome (OR652280.1). This ORF is highly conserved (≥98.46%, Cover 100%) in other *Medicago* species and shows significant similarity (94.72–97.12%, Cover 99%) to unannotated regions in *Lathyrus* and *Pisum* genera.

In silico translation of *orf279* revealed 100% identity of Orf279 to a hypothetical protein from *M. truncatula* (RHN66749.1). Structural modeling using SWISS-MODEL Workspace/GMQE confirmed its high similarity to maturase K (AlphaFold: A0A396IMA3.1.A). Further structural analysis of Orf279 identified its distant homology to a group II intron reverse transcriptase/maturase (PDB: 5hhj.1.B; 18.52% identity) and a CRISPR-associated Cas6-RT-Cas1 protein (PDB: 7kft.1.D; 18.64% similarity), suggesting a potential evolutionary link to mobile genetic elements.

In summary, we have characterized a novel and highly conserved mitochondrial ORF (*orf279*) that encodes a protein structurally homologous to maturase K, with additional remote similarities to reverse transcriptase and CRISPR-associated proteins.

#### 2.1.2. The Mosaic Nature of mtDNA Genes in VIK32, Line MlS-1

A mosaic structure (presence of introns and exons) was identified in eight protein-coding genes: *nad1*, *nad2*, *nad4*, *nad5*, *nad7*, *rps3*, *rps10*, and *ccmFc* (Table 1; Figure 2). The length of the exons in these genes ranged from 69 to 1562 bp. The distance between the first and last exons was 133 kb, 152 kb, and 215 kb for *nad1*, *nad2*, and *nad5*, respectively, while for the remaining six genes, this distance did not exceed 9 kb (Figure 2).

**Figure 4:**
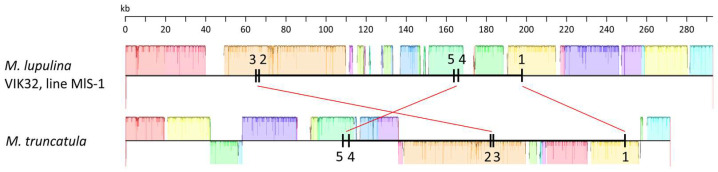
Localization of the *nad1* gene exons in the mtDNAs of *M. lupulina* VIK32, line MlS-1 and *M. truncatula*. Vertical black lines (1–5) represent exons (see text), horizontal black lines represent introns; red lines connect the corresponding exons, and colored blocks represent similar nucleotide sequences in the respective mtDNAs (mtDNA alignment performed using Mauve v20150226 build 10).

The number of exons and introns varied significantly among the genes. Specifically, the *rps3*, *rps10*, and *ccmFc* genes each contained two exons and one intron; the *nad4* gene had three introns and four exons; and the *nad1*, *nad2*, *nad5*, and *nad7* genes each had five exons and four introns (Figure 2 and Figure 4). The sequences of the corresponding exons in the VIK32, MlS-1 mtDNA were identical to those in the *M. truncatula* reference mtDNA (Cover 100%, Identity 100%). Exceptions were exon 2 of *rps3* and *nad5* (Identity 99.49% and 99.84%, respectively), exon 4 of the *nad1* gene (Identity 98%), and exon 1 of the *rps10* and *ccmFc* genes (Identity 99.2% and 99.87%, respectively), where SNPs were identified.

The relative arrangement of exons was similar for seven of the eight genes mentioned above in both the VIK32, line MlS-1 and *M. truncatula* mtDNAs (Figure 2 and Figure 4). The only exception was the *nad1* gene, where the order of exons differed between the MlS-1 and *M. truncatula* mtDNAs, namely 3-2-5-4-1 and 5-4-2-3-1, respectively (Figure 4).

### 2.2. Intraspecific Comparison of M. lupulina mtDNA

**Figure 5:**
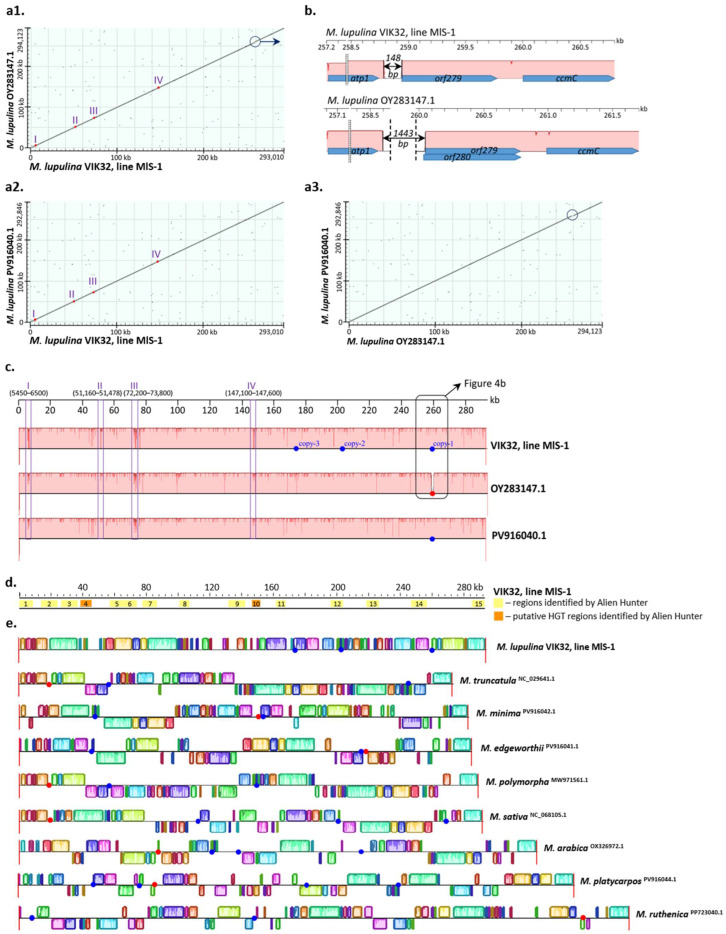
Alignments of mtDNAs of *M. lupulina* VIK32, line MlS-1 and wild-growing *Medicago* species. (**a**) Dot Plot of mtDNAs of VIK32, line MlS-1 and *M. lupulina* OY283147.1 and PV916040.1; subfigures (**a1**–**a3**) represent pairwise alignments (Dot Plots) between VIK32 and OY283147.1, VIK32 and PV916040.1, OY283147.1 and PV916040.1 mitogenomes, correspondingly; blue circles represent extended insertions/deletions (indels) in the mtDNA sequences; I–IV indicate regions with an increased frequency of substitutions in the studied mitogenomes (see text). Panels (**b**,**c**,**e**) show the Mauve alignment (Mauve v20150226 build 10) of the mtDNA sequences. (**b**) Regions of the VIK32, line MlS-1 mtDNA with 99–100% identity (pink blocks) and unique regions (colorless blocks). (**c**) Regions I-IV with an increased frequency of substitutions (see text) in the studied mitogenomes, which are also indicated in (**a**); blue circles—copies of the 148 bp sequence (see text); red circle—location of the 1443 bp sequence, which is shown in (**b**). (**e**) Mauve alignment of mitogenomes from 9 *Medicago* plants of different species (see Section 4: Materials and Methods); colored blocks—similar sequences in the mtDNAs of *Medicago* plants (Identity > 50%); blue circles—copies of the 148 bp sequence (see text); red circles—copies of the 1443 bp sequence (see text). Mauve alignment of 17 *Medicago* mitogenomes deposited in GenBank (see Section 4: Materials and Methods; Appendix A: Figure A1). (**d**) Regions of predicted horizontal gene transfer (HGT) in the VIK32, line MlS-1 mtDNA (yellow blocks).

The nucleotide sequences of the VIK32, line MlS-1 mtDNA and the mtDNAs of *M. lupulina* OY283147.1 or *M. lupulina* PV916040.1 showed a high degree of similarity (Cover 100%, Identity 99.52–99.86%; Appendix B: Table A2; Figure 5(a1–a3)). However, extended non-homologous sequences were identified (Figure 5b).

Furthermore, during a pairwise comparison of the three *M. lupulina* mtDNAs (VIK32, line MlS-1; OY283147.1; and PV916040.1), four regions with a high degree of polymorphism were identified. In the MlS-1 mtDNA, the average frequency of SNPs in these regions was 11.0 SNPs per 100 bp, which was more than 32 times higher than the average SNP frequency across the entire mtDNA sequence (0.34 SNPs per 100 bp) (regions I–IV; Figure 5a,c). The SNP frequencies relative to *M. lupulina* OY283147.1/PV916040.1 in these specified regions were 9.3/8.8, 10.1/16.7, 12.3/12.1, and 12.1/12.0 substitutions per 100 bp, respectively.

Regions I and III were identified in the central parts of the corresponding *rnl* and *rns* genes, which encode the large subunit ribosomal RNA and small subunit ribosomal RNA. Region I spanned 33% of the *rnl* gene length (1050 out of 3176 bp of the gene), and Region III occupied 79% of the *rns* gene length (1600 out of 2014 bp of the gene). The presence of these regions resulted in a low level of identity for the corresponding *rnl* genes in VIK32, line MlS-1 and both *M. lupulina* accessions (Cover 100%, Identity 96.86%;). In the case of the *rns* gene from the VIK32 mtDNA, the similarity level with the corresponding gene from both *M. lupulina* accessions (Cover 100%, Identity 90.1%;) was even lower and comparable to that of the corresponding gene from the mtDNA of the phylogenetically distant plant *Glycine soja* (Cover 100%, Identity 91.9%).

Region II, with a length of 318 bp, was identified in the central part of the gene annotated as *rpl2* (354 bp), which encodes the ribosomal protein L2 and shows similarity to the exon of the corresponding chloroplast gene (*rplB*; see above) [18].

Region IV is located in the intergenic space between *orf100_2* and *orf103_5* (coordinates 145,163–145,465 and 147,584–147,895, respectively).

Significant sequence differences were revealed between the mtDNAs of line MlS-1 and *M. lupulina* OY283147.1 in the region located between the *atp1* and *ccmC* genes (Figure 5b). In this region of the *M. lupulina* OY283147.1 mtDNA, two overlapping ORFs (*orf279* and *orf280*), encoding hypothetical proteins, were identified. In contrast, only *orf279* (coordinates 258,943–259,779) was annotated in the VIK32, MlS-1 mtDNA (both mtDNA sequences were annotated using MFannot (https://megasun.bch.umontreal.ca/apps/mfannot/, accessed 3 October 2025); Figure 5b).

The mtDNA of VIK32, line MlS-1, between the ORFs *atp1* and *orf279*, contains a 148 bp sequence (coordinates 258,793–258,941), whereas the mtDNA of *M. lupulina* OY283147.1 contains a sequence spanning 1443 bp (coordinates 258,604–260,047). No similarity was detected between the aforementioned sequences (Figure 5b). The 1443 bp sequence (GenBank accession: OY283147.1) from *M. lupulina* contains the start codon of *orf280*.

A sequence of 1443 bp was also identified in the mtDNAs of representatives from eight other *Medicago* species (Cover 34–99%; Identity 82–100%); however, its localization and genetic context differed (Figure 5e).

The 148 bp sequence was identified in three copies in the VIK32, line MlS-1 mtDNA (Cover 75–100%, Identity 96–99%) (Figure 5c). The second copy of this sequence was adjacent to the 5′ end of the *atp8* gene and had coordinates 201,629–201,776 (Cover 100%, Identity 99.3%) (Figure 5c). The third copy represented a fragment of this sequence located between *orf101_1* and *orf120_1*, with coordinates 173,974–174,084 (Cover 75%, Identity 96.4%) (Figure 5c).

In the mtDNAs of other plants from the *Medicago* genus, this sequence was also present in either three or two copies; however, it was located in different regions of the mitogenome (Figure 5e).

Therefore, homologous extended sequences in *Medicago* mitogenomes exhibit different localizations and/or orientations. This suggests that these sequences were involved in intra-mitogenomic rearrangement processes, which led to the emergence of mitochondrial nucleotide sequence polymorphism at both the genus and species levels.

### 2.3. Phylogenetic Analysis of mtDNAs

A phylogenetic analysis of the mtDNA of VIK32, line MlS-1 was performed relative to 17 mitogenomes from representatives of nine *Medicago* species (*M. lupulina*, *M. truncatula*, *M. minima*, *M. edgeworthii*, *M. polymorpha*, *M. sativa*, *M. arabica*, *M. platycarpos*, and *M. ruthenica*), and the mitogenome of *Trigonella foenum-graecum*.

The mitogenomes of two closely related species of the genus *Glycine* (*G. soja* and *G. max*), *Lotus japonicus*, *Pisum sativum*, *Vavilovia formosa*, and *Lathyrus sativus* were used as an outgroup (see Section 4: Materials and Methods).

**Figure 6:**
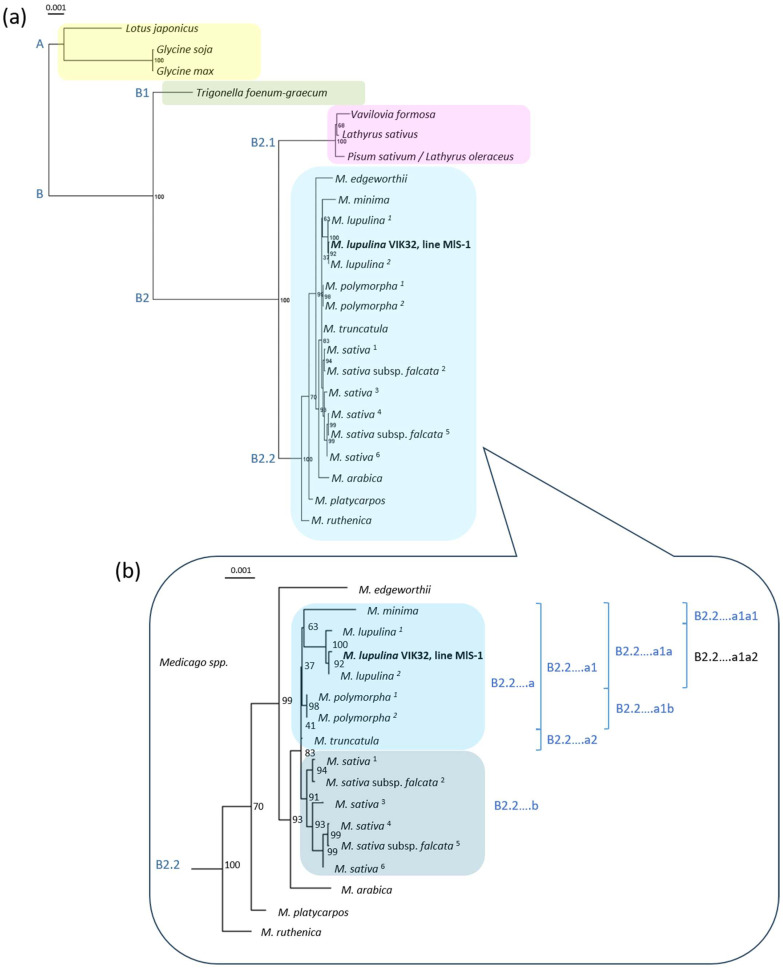
Phylogenetic analysis of mitochondrial genomes. (**a**) Phylogenetic tree of various legume species, including outgroups. A and B (B1, B2, B2.1, B2.2)—phylogenetic clusters and subclusters (see text). (**b**) Detailed phylogeny of *Medicago* species. The list of legume plants is given in text. GenBank accession numbers of mtDNAs of *Trigonella foenum-graecum*, *Glycine soja*, *G. max*, *Lotus japonicus*, *Pisum sativum*, *Vavilovia formosa*, and *Lathyrus sativus* see in Section 4: Materials and Methods, Section 4.4. *Medicago lupulina*: ^1^—OY283147.1, ^2^—PV916040.1; *M. polymorpha*: ^1^—MW971562.1, ^2^—MW971561.1; *M. sativa*: ^1^—NC_068105.1, ^2^—PV916043.1, ^3^—PV916045.1, ^4^—OR652281.1, ^5^—OQ612687.1, ^6^—OR652280.1. B2.2, B2.2….a –B2.2….a1a2 – phylogenetic subclusters, groups and subgroups, and branches (see text).

The phylogenetic tree was constructed based on the concatenated sequences of 23 mitochondrial genes, which we identified in all the plant species mentioned above (see Section 4: Materials and Methods). Two main clusters, A and B, were revealed (Figure 6a). Cluster A contained three mitogenomes: *G. soja*, *G. max*, and *L. japonicus*.

Cluster B was divided into two subclusters. Subcluster B1 included the mitogenome concatenate of *T. foenum-graecum*. Subcluster B2 was further divided, with subcluster B2.1 uniting the mitogenome concatenates of *V. formosa* (a relict legume), *L. sativus*, and *P. sativum*, the latter recently reclassified as *Lathyrus oleraceus*. The second subcluster, B2.2, united the concatenates of 21 mitogenomes from plants of nine *Medicago* species (Figure 6).

This robust clustering suggests that despite the high mosaic nature of the mitogenomes in this genus, functionally significant genes are conserved.

A downstream clustering analysis of subcluster B2.2 revealed that the mitogenomes of *M. ruthenica*, *M. edgeworthii*, *M. platycarpos*, and *M. arabica* formed distinct, separate branches not affiliated with larger sequence groups (Figure 6b).

The sequences of the *M. polymorpha* mitogenomes clustered into a single, highly supported group (bootstrap 98%), indicating a high degree of similarity among the studied sequences. In contrast, the mitogenomes of the four *M. sativa* and two *M. sativa* subsp. *falcata* representatives exhibited considerable diversity, forming group B2.2….b with high bootstrap support (91–99%). The close clustering of sequences from *M. sativa* and *M. sativa* subsp. *falcata* is consistent with the results from whole chloroplast genome sequence analysis [18] and with analyses of the nucleotide sequences of the internal transcribed ribosomal spacers (ITS1 and ITS2) and the external transcribed ribosomal spacers [25].

Overall, the analysis demonstrated a clear phylogenetic separation between the mitogenomes of *M. polymorpha* and the *M. sativa* species group. This result confirms the genetic cohesion within each taxon while simultaneously highlighting significant divergence between them.

The mitogenome sequences of *M. lupulina* and *M. minima* clustered into a distinct group, designated B2.2….a1a. A downstream clustering analysis of this group revealed that the *M. minima* mitogenome sequence formed a cluster with those of *M. lupulina* in 63% of bootstrap replicates (subgroups B2.2….a1a1 and B2.2….a1a2).

Within the *M. lupulina* subgroup, the mitogenome sequence of the studied line VIK32, line MlS-1 grouped together with the *M. lupulina* PV916040 sequence (bootstrap 92%), whereas the *M. lupulina* sequence OY283147.1 consistently formed a separate branch (bootstrap 100%). Consequently, the mitogenomes of the *M. lupulina* accessions from China and Kyrgyzstan (Central Asia)—the region of origin of the wild population from which the VIK32 breeding line MlS-1 was derived—were phylogenetically closer to each other than to the wild *M. lupulina* accession from Gorebridge, Scotland, UK.

These results are consistent with data obtained from the clustering analysis of complete chloroplast genome sequences of the same *M. lupulina* accessions [18] and with the analysis of three marker regions—the transcribed intergenic ribosomal spacers [25].

In summary, the analysis confirmed that the mitogenomes of *M. lupulina* and *M. minima* form a distinct clade (B2.2….a1a), which aligns with data from chloroplast and nuclear markers. Furthermore, within this clade, the *M. minima* mitogenome showed a variable position relative to *M. lupulina*, and a phylogenetically close subgroup, comprising accessions from China and Kyrgyzstan, was identified among the latter.

### 2.4. Synteny of mtDNAs in Legume Plants from Different Genera

An analysis of synteny was conducted on the mitogenomes of plants from different genera. The analysis included the mtDNA sequences of *M. lupulina* VIK32, line MlS-1, *Glycine max* (NC_020455.1), as well as the plants *Trigonella foenum-graecum* (NC_072135.1), *Lotus japonicus* (NC_016743.2), *Vavilovia formosa* (MK748602.1 and MK748603.1), *Lathyrus sativus* (PQ412513.1), and *Pisum sativum* (PP657342.1). Pairwise alignment of the MlS-1 mtDNA with the aforementioned legume plants was performed.

**Figure 7:**
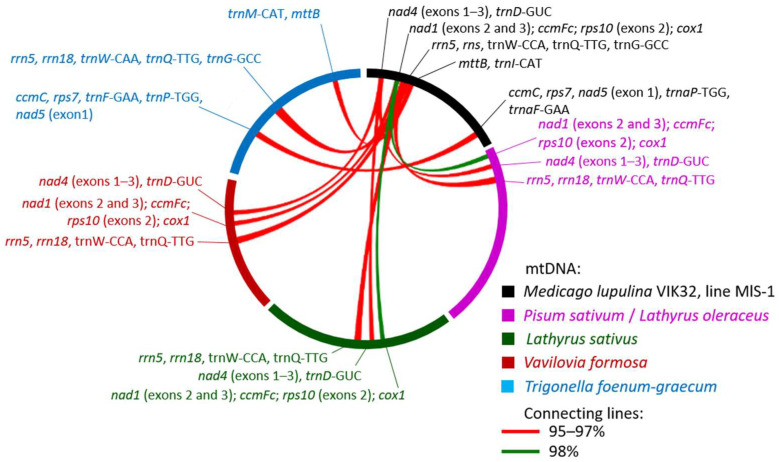
Synteny of sequences in the VIK32, line MlS-1 mtDNA and legume plants from four different genera. Genes within syntenic blocks were identified using annotations from the corresponding GenBank mitochondrial genomes. A summary of these blocks, including coordinates, percent identity, and annotated genes, is provided in Table A3. Detailed BLASTn (BLAST+ 2.17.0) alignment results are available in Table S2.

In total, 59 sequences longer than 9 kb and with an identity of ≥95% were identified among the different mtDNAs. Of these, 12 sequences were found to be similar between the VIK32, line MlS-1 mtDNA and the mtDNAs of four legume genera: *Trigonella foenum-graecum*, *Pisum sativum*, *Lathyrus sativus*, and *Vavilovia formosa* (MK748602.1) (Figure 7). As shown in Figure 7, the localization of these sequences differed across the mtDNAs.

Only one sequence was identified in plants from all four genera (Figure 7; Appendix B: Table A3). Two sequences with high identity were found in *Vavilovia formosa*, *Pisum sativum*, and *Lathyrus sativus*. Two other sequences showed similarity only with *Trigonella foenum-graecum* (Figure 7; Appendix B: Table A3).

It was shown that the identified sequences could contain both complete genes: *cox1*, *rrn5*, *rns*, *mttB*, *ccmC*, *rps7*, tRNA genes, as well as individual exons of the genes: *nad1*, *ccmFc*, *rps10*, *nad4*, *nad5* (Appendix B: Table A3).

Thus, despite the different clustering patterns (Figure 6a), extended similar sequences are present in the mitogenomes of plants from different genera. However, the majority of the mitogenomes exhibit a nucleotide sequence similarity level of less than 95%.

### 2.5. Potential HGT Candidates in the M. lupulina Mitogenome

A search for regions potentially introduced by horizontal gene transfer (HGT) identified 15 regions in the VIK32, line MlS-1 mtDNA using Alien Hunter v1.3.0 and none using PHASTEST v1.0.1. These regions range from 5 to 12.5 kb in length, with a total length equivalent to 124.7 kb or 42.6% of the VIK32 mtDNA size (Figure 2 and Figure 5d).

It was shown that 10 out of the 15 HGT regions (regions 1–3, 5–8, 11, 14, 15) were present in all 9 analyzed mitogenomes from plants of five different species: *M. lupulina*, *M. truncatula*, *M. sativa*, *M. polymorpha*, and *M. arabica* (Cover 98–100%). Furthermore, nearly all of them contained genes or gene fragments characteristic of all plant mtDNAs. Another 3 out of the 15 HGT regions (regions 9, 12, 13) identified in VIK32, line MlS-1 were present only fragmentarily in the analyzed plants.

Region 4 was entirely identified in the mtDNAs of *M. lupulina* (VIK32, line MlS-1; OY283147.1; and PV916040.1; Cover 100%, Identity 99.7%) (Figure 5d). Fragments of Region 4 were found in the mtDNAs of *M. polymorpha* and *M. arabica* (average values: Cover 52.6%, Identity 98.7%). This region was not detected in plants of other *Medicago* species. Region 4 contains one of three genes (*orf603*, coordinates 41,506–43,317), whose product is similar to the RdRp of mitoviruses (see above) and thus may potentially be of viral origin.

Region 10 was identified in its entirety only in the mtDNAs of *M. lupulina* (Cover 99–100%, Identity 99.5–100%), and partially in the mtDNAs of *M. arabica* (Cover 83%, Identity 99.4%), *M. polymorpha* MD013 and MD014 (Cover 64%, Identity 91.9%), and *M. sativa* Zhongmu-1 and Zhongmu-4 (average values: Cover 27%, Identity 99.5%). Region 10 was not detected in the mtDNA *M. sativa* NC_068105.1 or the reference mtDNA of *M. truncatula*.

This region contains a 603 bp fragment (gene *orf103_5*: 147,584–147,895), whose sequence is similar (Cover 100%, Identity 100%) to a fragment of the 23S rRNA gene located in the chloroplast of *Medicago* plants (see above), as well as in plants from phylogenetically distant genera (*Trifolium* and *Pisum*). Furthermore, this region partially overlaps with region IV, which is characterized by increased polymorphism (see above). The obtained data suggest that HGT region 10 was formed through the process of horizontal gene transfer from the chloroplast.

Thus, it is likely that regions 1–3, 5–9, and 11–15 were probably not introduced via HGT but could have been involved in intra-mitogenomic rearrangements. Region 4 was potentially introduced with the involvement of a *Mitoviridae* sp. phage. Region 10 may potentially have been acquired through horizontal gene transfer from the chloroplast.

## 3. Discussion

Traditionally, intraspecific variation in mitochondrial DNA (mtDNA) has been considered selectively neutral, with its role in the evolution of complex phenotypes such as lifespan or stress tolerance deemed insignificant [26]. However, accumulating evidence in the literature convincingly demonstrates that mtDNA can be subject to strong evolutionary selection and be directly involved in shaping life-history strategies [15]. Our analysis of the *M. lupulina* VIK32, line MlS-1 mitogenome revealed significant polymorphism and unique structural features of the line. We identified instances of intraspecific polymorphism, mosaic gene architecture at the genus level, and the presence of intriguing sequences potentially acquired via horizontal gene transfer, all of which point to the dynamic evolution of mitochondrial DNA in plants of the genus *Medicago*.

The key result of our study is the detailed description of the mosaic structure of the *M. lupulina* VIK32, line MlS-1 mitogenome, containing syntenic blocks with mitogenomes of plants of other species and/or genera, as well as with the chloroplast genome, interspersed with unique sequences. Comparative analysis of three *M. lupulina* mitogenomes (VIK32, line MlS-1, PV916040.1 and OY283147.1) showed high identity of their common sequences, but revealed significant differences, among which extended indels and specific hypervariable regions inside functionally important genes, such as *rnl* and *rns*, which encode the large subunit ribosomal RNA and small subunit ribosomal RNA. The complex mosaic structure of eight out of 33 core genes, especially the *nad1* locus encoding the NADH dehydrogenase subunit, has been identified. In the case of the *nad1* gene in the VIK32, line MlS-1 mitogenome, SNPs potentially affecting protein function have been identified, and it has also been shown that the exon distribution of *nad1* is species-specific for *M. lupulina*, in contrast to the model organism *M. truncatula*. All of this points to a significant role of the mutational process and recombination in the evolution of mitogenomes, which has also been noted in the works of other authors [15,27].

All identified structural changes in the mitogenome contribute to the observed genus-level diversity demonstrated in the phylogenetic analysis. Our phylogenetic reconstruction firmly places *M. lupulina* and *M. minima* into a distinct, well-supported clade within the genus *Medicago*, which is consistent with the data from [28] and is supported by data obtained from the study of chloroplast and nuclear markers [18,25]. The contrasting patterns observed between species—for instance, the high conservation in *M. polymorpha* compared to the significant diversity within the *M. sativa* spp. complex—illustrate that even closely related taxa can differ substantially in their mitogenome evolutionary rates. The synteny analysis further shows that, despite species-specific variations, a common “core of functionally significant genes” is conserved among different legume species of the genus *Medicago*.

An important finding is the identification of clearly distinct mitotypes that correlate with geographic origin—specifically, the clustering of line VIK32 (Kyrgyzstan/Central Asia) with PV916040 (China) and their distinction from the sample OY283147.1 from Scotland, UK. This mitogenomic variation, consistent with our previous data on the chloroplast genome analysis of the same *M. lupulina* samples, provides a basis for investigating potential links between mitochondrial haplotypes and adaptive traits, such as the dwarfism of the MlS-1 line and its dependence on phosphorus nutrition [23].

The genetic diversity of chloroplasts and mitochondria, as energy-producing organelles, and their interplay with nuclear genetic material, which has become the focus of active research in recent years [15,29,30,31,32], also presents significant scientific interest for understanding the observed morpho-phenotypic diversity of *M. lupulina*. This is particularly relevant for elucidating its unique ability to adapt to various environmental conditions [19,20].

A testament to the dynamic evolutionary history of the mitochondrial genome is the identification of sequences exhibiting a high degree of similarity with bacterial genes. The VIK32 mitogenome serves as a remarkable archive of sequences tracing back to its bacterial ancestor, potentially acquired through horizontal gene transfer. A conserved open reading frame (ORF), *orf279*, widespread in *Medicago* mitogenomes yet showing distant similarity to bacterial enzymes, emerges as a promising candidate for a novel, functionally important gene that may have been “domesticated” following a horizontal transfer event. The finding of ORFs demonstrating significant homology to maturase/reverse transcriptase from group II introns and, notably, to CRISPR-associated proteins (e.g., Cas1, Cas6-RT-Cas1), suggests a complex history of acquisition for these genes.

The presence of conserved tRNA genes, common to both mitochondria and chloroplasts, exhibits similarity to corresponding genes in cyanobacteria. The presence of a fragment within the rRNA gene, which showed high similarity to those in *Rhodospirillum rubrum* (a relative of the mitochondrial progenitor), represents traces of an “ancient genetic record” from the ancestral protomitochondrion.

Moreover, the mitogenome contains sequences such as *orf603*, which resembles RNA-dependent RNA polymerases of mitoviruses, and *orf103_5*, which shows high similarity to a fragment of the chloroplast 23S rRNA, points to a connection between plant organelles. Further evidence is provided by the localization of *orf103_5* within a predicted HGT (Horizontal Gene Transfer) region (Region 10). These findings strongly support the concept of an ongoing evolutionary dialog between the two organelles and serve as additional evidence for the prokaryotic origin of plant organelles.

In conclusion, the characterization of the *M. lupulina* MlS-1 mitogenome significantly expands and deepens our understanding of organelle evolution in legumes. Our work establishes a foundation for further investigation into the link between structural changes in the mitochondrial genome and key agronomically significant phenotypes. The dwarfism and high symbiotic responsiveness of the MlS-1 line can be further explored in the context of correlating mitotypes with stress resistance in *M. lupulina*. This makes black medick a promising model for studying the role of bacterial-origin organelles in improving the yield potential of economically valuable plant species.

## 4. Materials and Methods

### 4.1. Obtaining the Plant Material for Analysis

Plants of *Medicago lupulina* L. var. *vulgaris* Koch, line MlS-1 from the cultivar-population VIK32, were obtained through selective breeding for high responsiveness to symbiosis with the arbuscular mycorrhizal fungus (*Rhizophagus irregularis*) [23]. In addition to this selected trait, plants of this line retained the ability to form an effective symbiosis with root nodule bacteria of the genus *Sinorhizobium*. The symbiotic performance of this line with *Ensifer meliloti* strain L6-AK89 was comparable to that of the parent cultivar-population VIK32, as indicated by our analysis showing a non-significant difference in dry weight of inoculated plants (*p* > 0.05; see Appendix A).

Seeds of the annual diploid *Medicago lupulina* L. var. *vulgaris* Koch (cultivar-population VIK32, line MlS-1 [23]) were surface-sterilized in concentrated sulfuric acid for 10 min, rinsed five times with distilled water, and germinated on moist, sterile vermiculite in Petri dishes at 28 °C for 3 days. Seedlings were grown individually in sterile tubes containing vermiculite and irrigated with the liquid Krasilnikov-Korenyako nutrient medium [33]. Plants were maintained at 25–26 °C under an 18/6-h light/dark photoperiod (Versatile Environmental Test Chamber MLR-351H; Sanyo, Osaka, Japan). Leaves from a single plant were flash-frozen in liquid nitrogen and stored at –80 °C.

### 4.2. DNA Sequencing and Assembly

Plant genomic DNA was isolated using a modified cetyltrimethylammonium bromide (CTAB) protocol [34]. The CTAB buffer was supplemented with 3% polyvinylpyrrolidone (PVP) and 3% β-mercaptoethanol (Sigma-Aldrich, St. Louis, MO, USA). Organic phase separation was repeated until the aqueous phase was clear. The DNA pellets were dissolved in 200 μL of DNase-free water, treated with RNase A (Thermo Fisher Scientific, Waltham, MA, USA), and subjected to another round of chloroform purification. DNA was recovered by ethanol precipitation, resuspended in DNase-free water, and stored at –20 °C.

A paired-end (PE) library was prepared with dual-index NEBNext multiplex oligonucleotides and the NEBNext Ultra II DNA Library Prep Kit for Illumina (New England Biolabs, Ipswich, MA, USA) and sequenced on an Illumina HiSeq 2500 platform at Macrogen Europe (Amsterdam, Netherlands). Long-read sequencing was performed on a MinION device (Oxford Nanopore Technologies, Oxford, UK) at the Federal State Budget Scientific Institution “All-Russia Research Institute for Agricultural Microbiology” (FSBSI ARRIAM). The assembly was conducted using Flye v. 2.9. Default parameters were used for all software.

### 4.3. Mitochondrial DNA Analysis

Mitochondrial genes were annotated using MFannot (https://megasun.bch.umontreal.ca/apps/mfannot/, accessed 3 October 2025) [35] and Proksee (https://proksee.ca/, accessed 3 September 2025) [36] with the Standard Genetic Code. The initial annotations were manually verified using BLASTn and BLASTp (BLAST+ 2.17.0) [37,38]. A targeted search was conducted for genes known to be present in the *Medicago truncatula* mitogenome—a well-established model for legume biology [16,17]—focusing on exons and introns in genes with BLASTn (BLAST+ 2.17.0). Transfer RNA (tRNA) genes were identified using Aragorn v1.2.41 [39].

Similar direct and inverted repeats in the mitochondrial DNA were identified using Mauve v20150226 build 10 [40]. All mitogenomes in the alignment were reoriented to start from the same point as the *Medicago truncatula* reference mitogenome (NC_029641.1).

A circular map of the mitochondrial genome was generated with Proksee (https://proksee.ca/, accessed 3 September 2025) [36], which was used to visualize GC Skew, GC Content (nucleotide composition), and to run Alien Hunter v1.3.0 for the identification of atypical sequences. Additional analyses included a search for prophage-like regions using PHASTEST v1.0.1 [41] and the identification of CRISPR-Cas components using CRISPRCasFinder v4.2.20 [42] within Proksee. Default parameters were used for all software.

A comparative dot plot of the VIK32, line MlS-1 and wild-growing *M. lupulina* mitogenomes was generated using BLASTn (BLAST+ 2.17.0).

The relative rate of substitution accumulation (N) between sequences was calculated using the formula: N = (L/100) × *n*, where L is the sequence length and *n* is the number of substitutions per 100 nucleotides.

The primary sequence and 3D-structure of the hypothetical proteins were analyzed using BLASTp (BLAST+ 2.17.0), InterProScan v5.76-107.0 [43], and SWISS-MODEL Workspace / GMQE [44] tools.

### 4.4. Phylogenetic Analysis

For the phylogenetic analysis, we selected nine *Medicago* plants and seven other *Fabaceae* species (Table 2). Twenty-one conserved mitochondrial protein-coding genes (*atp1*, *atp4*, *atp6*, *atp8*, *atp9*, *cob*, *cox1*, *cox3*, *rps12*, *nad1*, *nad2*, *nad3*, *nad4*, *nad4L*, *nad5*, *nad6*, *nad7*, *nad9*, *matR*, *ccmB*, *ccmFc*, *ccmFn*, and *ccmC*) were concatenated to construct the phylogenetic tree. For the genes *nad1*, *nad2*, *nad4*, *nad5*, *nad7*, and *ccmFc*, only the exon sequences were used.

### 4.5. Phylogenetic Tree Construction

Multiple nucleotide sequence alignment was performed with MAFFT v7.450 using the UPGMA algorithm [55]. Phylogenetic relationships were inferred using IQ-TREE v3 [56] under the maximum likelihood criterion with 10,000 bootstrap replicates. The resulting tree was visualized and midpoint-rooted in Dendroscope v3.8.10 [57].

## Figures and Tables

**Table 1:** Genes encoding proteins with predicted function in the mtDNA of *M. lupulina* VIK32, line MlS-1.

Protein Coding Genes		Groups of Genes with Predicted Function
Without Introns	With Introns	
*nad3*, *nad4L*, *nad6*, *nad9*	*nad1*, *nad2*, *nad4*, *nad5*, *nad7*	Complex I ^1^
*cob*	- ^2^	Complex III ^1^
*cox1*, *cox2*, *cox3*	-	Complex IV ^1^
*atp1*, *atp4*, *atp6*, *atp8*, *atp9*	-	Complex V ^1^
*ccmC*, *ccmFn*, *ccmB*	*ccmFc*	Cytochrome c maturation
*rps4*, *rps7*, *rps12*, *rps14*	*rps3*, *rps10*	Ribosomal protein small subunit
*rpl2*, *rpl5*, *rpl16*	-	Ribosomal protein large subunit
*matR*	-	Processing and maturation of various pre-RNAs
*mttB*	-	Transport membrane protein
Total: 25 genes	8 genes	9 groups

^1^—Complex I—NADH dehydrogenase; Complex III—cytochrome c reductase/cytochrome bc1 complex; Complex IV—cytochrome c oxidase; Complex V—ATP synthase. ^2^—no genes with such characteristics have been identified.

**Table 2:** Nucleotide sequences of mitochondria used in the study.

Plant Genus	Plant Species	Source	Length, bp	GC Content, %	GenBank Accession * or/and Reference	Submission Date
*Medicago*	*M. lupulina* L.	var. *vulgaris* Koch, cultivar-population VIK32, line MlS-1 (this study)	293,010	45.3	///	2025
		specimen from Gorebridge, Scotland, UK (Royal Botanic Garden Edinburgh; BioSample: SAMEA11478582)	294,123	45.4	OY283147.1, [45]	2023
		-	292,846	45.4	PV916040.1, [28]	2025
	*M. polymorpha*	voucher I.S. Choi MD014 (TEX-LL)	287,639	45.3	MW971562.1, [46]	2021
		voucher I.S. Choi MD013 (TEX-LL)	287,636	45.3	MW971561.1, [46]	2021
	*M. truncatula*	A17	271,618	45.4	NC_029641.1, [17]	2015
	*M. sativa*	isolate Zhongmu-4 cultivar Zhongmu-4	306,983	45.1	OR652281.1, [1]	2023
		isolate Zhongmu-1 cultivar Zhongmu-1	299,123	45.3	OR652280.1, [1]	2023
		collected from China: Qinghai	290,285	45.3	NC_068105.1	2022
		-	297,911	45.4	PV916045.1, [28]	2025
		subsp. *falcata*, collected from Hohhot, Inner Mongolia, China	307,026	45.1	OQ612687.1, [47]	2023
		subsp. *falcata*	356,577	45.3	PV916043.1, [28]	2025
	*M. arabica*	specimen collected from Kingston Upon Thames, Surrey, UK	324,468	44.9	OX326972.1, [48]	2022
	*M. minima*	seeds were obtained from the Kunming Institute of Botany, Chinese Academy of Sciences	281,240	45.3	PV916042.1, [28]	2025
	*M. edgeworthii*	-	283,861	45.6	PV916041.1 [28]	2025
	*M. ruthenica*	collected from Hohhot, Inner Mongolia, China	354,988	45.1	PP723040.1, [49]	2024
	*M. platycarpos*	-	348,112	45.0	PV916044.1, [28]	2025
*Glycine*	*G. max*	Aiganhuang (N21249), a typical landrace grown in the Huang-Huai river valleys of China	402,558	45.0	NC_020455.1 [50]	2013
	*G. soja*	wild soybean; seeds were received from the National GeneBank of the Rural Development Administration of the Republic of Korea	402,545	45.0	NC_039768.1, [51]	2017
*Vavilovia*	*V. formosa*	*V. formosa* population in the North Ossetian State Natural Reserve, North Ossetia, the Caucasus, Russia	88,581 and 264,766	44.8 and 45.3	MK748603.1 and MK748602.1 [52]	2019
*Lotus*	*L. japonicus*	ecotype MG-20	380,861	45.4	NC_016743.2, [53]	2012
*Trigonella*	*T. foenum-graecum*	was cultivated at the medicinal herb planting base of the College of Pharmacy, Qinghai Minzu University (Xining, Qinghai, China).	345,604	45.3	NC_072135.1, [54]	2022
*Lathyrus*	*L. sativus*	-	379,804	45.3	PQ412513.1	2024
	*L. oleraceus* *(Pisum sativum)*	-	363,821	45.1	PP657342.1	2024

* accessed September 2025.

**Figure A1:**
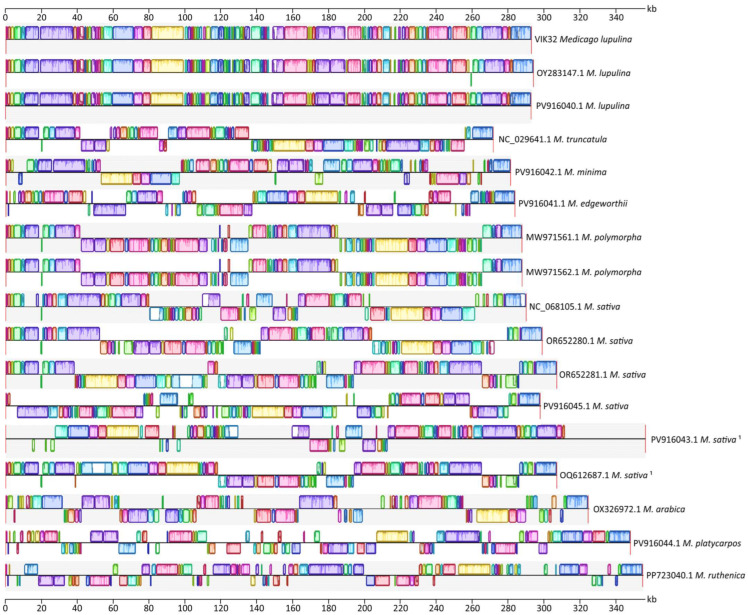
Whole-genome alignment of 17 *Medicago* mitogenomes retrieved from GenBank (October 2025), generated using Mauve v20150226 build 10. Colored blocks—similar sequences in the mtDNAs of *Medicago* plants (Identity > 50%). ^1^—*Medicago sativa* subsp. *falcata*.

## Data Availability

The original contributions presented in this study are included in the article/Supplementary Material. Further inquiries can be directed to the corresponding author.

## Supplementary Materials

The following supporting information can be downloaded at: https://www.mdpi.com/article/10.3390/ijms262411830/s1.

## Abbreviations

The following abbreviations are used in this manuscript:

mitogenome	Mitochondrial genome
mtDNA	Mitochondrial genome/Mitochondrial DNA
VIK32, line MlS-1	*Medicago lupulina* L. var. *vulgaris* Koch, cultivar-population VIK32, line MlS-1
HGT	Horizontal Gene Transfer
kb	kilobase, thousand base pairs
Mb	megabase, million base pairs
bp	base pair
GC content	guanine-cytosine content
ORF	open reading frame
SNP	single nucleotide polymorphism
rRNA	ribosomal RNA
tRNA	transfer RNA

## Appendix A. Plant Experiments

To assess the symbiotic efficiency of *M. lupulina* line MlS-1 with nodule bacteria of the genus *Sinorhizobium*, sterile microvegetative experiments were conducted. Seeds from the parental cultivar-population VIK32 from the collection of the V.V. Williams Federal Research Center of Forage Production and Agroecology (Lobnya, Moscow Region, Russia) were used as a control for symbiotic effectiveness. A genetically characterized native strain, *Ensifer* (*Sinorhizobium*) *meliloti* strain L6-AK89 [22], was used as a positive inoculation control. Non-inoculated plants of each studied symbiotic combination served as a negative inoculation control (NIC). Each “plant × rhizobial strain” combination had a technical replication of 10, as did the NIC for each combination. Plants were grown in sterile microvegetative experiments under a 16-h photoperiod (16,000 lux, alternating temperatures of 22 °C and 16 °C; Versatile Environmental Test Chamber MLR-351H; Sanyo, Osaka, Japan) for 56 days, as described previously [22,33]. Three biological replicates were performed. The symbiotic effectiveness of the studied combinations was evaluated based on the increase in plant biomass, measured after 56 days of growth, and calculated by subtracting the corresponding yield index (100%) according to [22]. Data were statistically processed by analysis of variance (ANOVA) using Microsoft Office Excel 2007.

The mean dry weight for symbiotic combinations formed by L6-AK89 and *M. lupulina* line MlS-1 was 21.667 ± 6.386 mg/plant, or 59.753 ± 27.082%, while for similar combinations with the parental cultivar-population VIK32, it was approximately the same (19.662 ± 3.962 mg/plant, or 59.416 ± 26.801%). The differences were not statistically significant (*p* > 0.05). Thus, the symbiotic efficiency with rhizobia in *M. lupulina* line MlS-1 did not differ from that of the parental cultivar-population VIK32.

**Table A1:** Evaluation of symbiosis between *Medicago lupulina* and *Ensifer meliloti* strain L6-AK89.

Plants	Parental VIK32		VIK32, Line MlS-1	
variant of inoculation	plant dry mass ± SE ^2^, mg/plant	% relative to control without inoculation	plant dry mass ± SE, mg/plant	% relative to control without inoculation
NIC ^1^	12,333 ± 2002	100 ± 12,599	13,563 ± 2501	100 ± 18,438
*E.* (*S.*) *meliloti* L6-AK89	19,662 ± 3962	159,416 ± 26,801	21,667 ± 6386	159,753 ± 27,082

^1^ NIC—negative inoculation control; ^2^ SE—standard error.

## Appendix B

**Table A2:** *Medicago lupulina* mtDNAs alignment.

*M. lupulina* Alignment *		Identity (%)		
		VIK32, Line MlS-1	OY283147.1	PV916040.1
Cover (%)	VIK32, line MlS-1	-	99.64	99.77
	OY283147.1	100	-	99.87
	PV916040.1	100	100	-

*—according to BLASTn (BLAST+ 2.17.0).

**Table A3:** Synteny analysis of the *M. lupulina* VIK32, line MlS-1 mitogenome with related legume species. The table lists the coordinates, percent identity, and annotated genes for each conserved syntenic block.

VIK32, Line MlS-1 mtDNA		Identity (%)			
Coordinates	Genes	*V. formosa* (MK748602.1)	*P. sativum* (PP657342.1)	*L. sativus* (PQ412513.1)	*T. foenum-graecum* (NC_072135.1)
27,166–36,562	3 exons of *nad4*, tRNA-D(GUC); 1 hypothetical protein	97.4	97.5	97.6	-
60,275–69,607 ^1^	2 exons of *nad1*; *ccmFc*; 1 exon of *rps10*; *cox1*	97.9	98.1	98.0	-
71,774–85,281 ^2,3^	*rrn5*, *rns*, tRNA-W(CCA), tRNA-Q(UUG), tRNA-G(GCC) ^3^	95.7	95.1	95.7	95.8
90,026–100,430	*mttB*; tRNA-M(CAU)	-	-	-	96.5
258,937–271,279	*ccmC*, *rps7*, 1 exon of *nad5*, tRNA-P(UGG), tRNA-F(GAA); 6 hypothetical proteins	-	-	-	96.5

The analysis identified: ^1^ a sequence similar to *Lathyrus sativus* beginning at position 60,499; ^2^ a sequence similar to *Vavilovia formosa* beginning at position 71,771; and ^3^ relevant data for *Trigonella foenum-graecum* 71,683–90,035.

## References

[1] He X., Zhang X., Deng Y., Yang R., Yu L.-X., Jia S., Zhang T. (2023). Structural Reorganization in Two Alfalfa Mitochondrial Genome Assemblies and Mitochondrial Evolution in *Medicago* Species. Int. J. Mol. Sci..

[2] Wu Z., Yang T., Qin R., Liu H. (2023). Complete Mitogenome and Phylogenetic Analysis of the *Carthamus tinctorius* L.. Genes.

[3] Schwartz R.M., Dayhoff M.O. (1978). Origins of Prokaryotes, Eukaryotes, Mitochondria, and Chloroplasts: A Perspective Is Derived from Protein and Nucleic Acid Sequence Data. Science.

[4] Panov A.V., Golubenko M.V., Darenskaya M.A., Kolesnikov S.I. (2020). The Origin of Mitochondria and their Role in the Evolution of Life and Human Health. Acta Biomed. Sci..

[5] Sørensen M.E.S., Stiller M.L., Kröninger L., Nowack E.C.M. (2025). Protein Import into Bacterial Endosymbionts and Evolving Organelles. FEBS J..

[6] Skippington E., Barkman T.J., Rice D.W., Palmer J.D. (2015). Miniaturized Mitogenome of the Parasitic Plant *Viscum Scurruloideum* Is Extremely Divergent and Dynamic and Has Lost All *Nad* Genes. Proc. Natl. Acad. Sci. USA.

[7] Putintseva Y.A., Bondar E.I., Simonov E.P., Sharov V.V., Oreshkova N.V., Kuzmin D.A., Konstantinov Y.M., Shmakov V.N., Belkov V.I., Sadovsky M.G. (2020). Siberian Larch (*Larix sibirica* Ledeb.) Mitochondrial Genome Assembled Using Both Short and Long Nucleotide Sequence Reads Is Currently the Largest Known Mitogenome. BMC Genom..

[8] Kozik A., Rowan B.A., Lavelle D., Berke L., Schranz M.E., Michelmore R.W., Christensen A.C. (2019). The Alternative Reality of Plant Mitochondrial DNA: One Ring Does Not Rule Them All. PLoS Genet..

[9] Gualberto J.M., Newton K.J. (2017). Plant Mitochondrial Genomes: Dynamics and Mechanisms of Mutation. Annu. Rev. Plant Biol..

[10] Unseld M., Marienfeld J.R., Brandt P., Brennicke A. (1997). The Mitochondrial Genome of *Arabidopsis thaliana* Contains 57 Genes in 366,924 Nucleotides. Nat. Genet..

[11] Sugiyama Y., Watase Y., Nagase M., Makita N., Yagura S., Hirai A., Sugiura M. (2005). The Complete Nucleotide Sequence and Multipartite Organization of the Tobacco Mitochondrial Genome: Comparative Analysis of Mitochondrial Genomes in Higher Plants. Mol. Genet. Genom..

[12] Møller I.M., Rasmusson A.G., Van Aken O. (2021). Plant Mitochondria—Past, Present and Future. Plant J..

[13] Monzel A.S., Enríquez J.A., Picard M. (2023). Multifaceted Mitochondria: Moving Mitochondrial Science beyond Function and Dysfunction. Nat. Metab..

[14] Wynn E.L., Christensen A.C. (2019). Repeats of Unusual Size in Plant Mitochondrial Genomes: Identification, Incidence and Evolution. G3.

[15] Mahapatra K., Banerjee S., De S., Mitra M., Roy P., Roy S. (2021). An Insight Into the Mechanism of Plant Organelle Genome Maintenance and Implications of Organelle Genome in Crop Improvement: An Update. Front. Cell Dev. Biol..

[16] Young N.D., Debellé F., Oldroyd G.E.D., Geurts R., Cannon S.B., Udvardi M.K., Benedito V.A., Mayer K.F.X., Gouzy J., Schoof H. (2011). The *Medicago* Genome Provides Insight into the Evolution of Rhizobial Symbioses. Nature.

[17] Bi C., Wang X., Xu Y., Wei S., Shi Y., Dai X., Yin T., Ye N. (2016). The Complete Mitochondrial Genome of *Medicago truncatula*. Mitochondrial DNA B Resour..

[18] Vladimirova M.E., Pernak E.V., Muntyan V.S., Saksaganskaia A.S., Kozlova A.P., Afonin A.M., Yurkov A.P., Zhukov V.A., Roumiantseva M.L. (2023). Chloroplast Genome of *Medicago lupulina* L. Var. *Vulgaris* Koch: Structure, Sequences Introduced as a Result of HGT and Viral Nature. Russ. J. Plant Physiol..

[19] Ahatović Hajro A., Hasanović M., Murtić S., Kalajdžić A., Pojskić N., Durmić-Pašić A. (2024). Serpentine Environment Prevails over Geographic Distribution in Shaping the Genetic Diversity of *Medicago lupulina* L.. Mol. Genet. Genom..

[20] Stepanova G.V. (2009). The Economic Value of Wild-Growing Samples of Black Medick (*Medicago lupulina* L.) of Various Ecological and Geographical Origins. Proc. Appl. Bot. Genet. Breed..

[21] Kosolapov V.M., Shamsutdinov Z.S., Ivshin G.I., Kuleshov G.F., Novoselov M.Y., Piskovatsky Y.M., Tyurin Y.S., Kharkov Y.D., Shamsutdinov N.E. (2015). Main Species and Varieties of Forage Crops: Results of the Scientific Activity of the Central Selection Center.

[22] Roumiantseva M.L., Vladimirova M.E., Saksaganskaia A.S., Muntyan V.S., Kozlova A.P., Afonin A.M., Baturina O.A., Simarov B.V. (2022). *Ensifer meliloti* L6-AK89, an Effective Inoculant of *Medicago lupulina* Varieties: Phenotypic and Deep-Genome Screening. Agronomy.

[23] Yurkov A.P., Jacobi L.M. (2018). *Medicago lupulina* lines with defects in the development of efficient arbuscular mycorrhiza. Ecol. Genet..

[24] Ezawa T., Silvestri A., Maruyama H., Tawaraya K., Suzuki M., Duan Y., Turina M., Lanfranco L. (2023). Structurally Distinct Mitoviruses: Are They an Ancestral Lineage of the *Mitoviridae* Exclusive to Arbuscular Mycorrhizal Fungi (Glomeromycotina)?. mBio.

[25] Bena G. (2001). Molecular Phylogeny Supports the Morphologically Based Taxonomic Transfer of the “Medicagoid” *Trigonella* Species to the Genus *Medicago* L.. Plant Syst. Evol..

[26] Stojković B., Sayadi A., Đorđević M., Jović J., Savković U., Arnqvist G. (2017). Divergent Evolution of Life Span Associated with Mitochondrial DNA Evolution. Evolution.

[27] Wang J., Zou Y., Mower J.P., Reeve W., Wu Z. (2024). Rethinking the Mutation Hypotheses of Plant Organellar DNA. Genom. Commun..

[28] Yang R., Wang M., Wang M., Li J., Li J., Huang C.-H. (2025). Assembly and Comparative Analysis of Chromosomal Mitochondrial Genomes in Multiple *Medicago* Species. BMC Plant Biol..

[29] Grabelnykh O.I. (2005). Energy functions of plant mitochondria under stress conditions. J. Stress Physiol. Biochem..

[30] Locato V., Cimini S., De Gara L. (2018). ROS and Redox Balance as Multifaceted Players of Cross-Tolerance: Epigenetic and Retrograde Control of Gene Expression. J. Exp. Bot..

[31] Jurdak R., Launay-Avon A., Paysant-Le Roux C., Bailly C. (2021). Retrograde Signalling from the Mitochondria to the Nucleus Translates the Positive Effect of Ethylene on Dormancy Breaking of *Arabidopsis thaliana* Seeds. New Phytol..

[32] Gupta M., Sharma G., Saxena D., Budhwar R., Vasudevan M., Gupta V., Gupta A., Gupta R., Chandran D. (2020). Dual RNA-Seq Analysis of *Medicago truncatula* and the Pea Powdery Mildew *Erysiphe pisi* Uncovers Distinct Host Transcriptional Signatures during Incompatible and Compatible Interactions and Pathogen Effector Candidates. Genomics.

[33] Roumiantseva M.L., Simarov B.V., Onishchuk O.P., Andronov E.E., Chizhevskaya E.P., Belova V.S., Kurchak O.N., Muntyan A.N., Rumyantseva T.B., Zatovskaya T.V. (2011). Biodiversity of Rhizobia in Ecosystems and Agrocenoses: Theoretical Bases and Methods.

[34] Doyle J.J., Doyle J.L. (1987). A Rapid DNA Isolation Procedure for Small Quantities of Fresh Leaf Tissue. Phytochem. Bull..

[35] Lang B.F., Beck N., Prince S., Sarrasin M., Rioux P., Burger G. (2023). Mitochondrial Genome Annotation with MFannot: A Critical Analysis of Gene Identification and Gene Model Prediction. Front. Plant Sci..

[36] Grant J.R., Enns E., Marinier E., Mandal A., Herman E.K., Chen C., Graham M., Van Domselaar G., Stothard P. (2023). Proksee: In-Depth Characterization and Visualization of Bacterial Genomes. Nucleic Acids Res..

[37] Samal K.C., Sahoo J.P., Behera L., Dash T. (2021). Understanding the BLAST (Basic Local Alignment Search Tool) Program and a Step-by-Step Guide for Its Use in Life Science Research. Bhartiya Krishi Anusandhan Patrika.

[38] Camacho C., Boratyn G.M., Joukov V., Vera Alvarez R., Madden T.L. (2023). ElasticBLAST: Accelerating Sequence Search via Cloud Computing. BMC Bioinform..

[39] Laslett D. (2004). ARAGORN, a Program to Detect tRNA Genes and tmRNA Genes in Nucleotide Sequences. Nucleic Acids Res..

[40] Darling A.C.E., Mau B., Blattner F.R., Perna N.T. (2004). Mauve: Multiple Alignment of Conserved Genomic Sequence with Rearrangements. Genome Res..

[41] Wishart D.S., Han S., Saha S., Oler E., Peters H., Grant J.R., Stothard P., Gautam V. (2023). PHASTEST: Faster than PHASTER, Better than PHAST. Nucleic Acids Res..

[42] Couvin D., Bernheim A., Toffano-Nioche C., Touchon M., Michalik J., Néron B., Rocha E.P.C., Vergnaud G., Gautheret D., Pourcel C. (2018). CRISPRCasFinder, an Update of CRISRFinder, Includes a Portable Version, Enhanced Performance and Integrates Search for Cas Proteins. Nucleic Acids Res..

[43] Blum M., Andreeva A., Florentino L.C., Chuguransky S.R., Grego T., Hobbs E., Pinto B.L., Orr A., Paysan-Lafosse T., Ponamareva I. (2025). InterPro: The Protein Sequence Classification Resource in 2025. Nucleic Acids Res..

[44] Waterhouse A., Bertoni M., Bienert S., Studer G., Tauriello G., Gumienny R., Heer F.T., de Beer T.A.P., Rempfer C., Bordoli L. (2018). SWISS-MODEL: Homology Modelling of Protein Structures and Complexes. Nucleic Acids Res..

[45] Ruhsam M., Royal Botanic Garden Edinburgh Genome Acquisition Lab, Darwin Tree of Life Barcoding Collective, Plant Genome Sizing Collective, Wellcome Sanger Institute Tree of Life Management, Samples and Laboratory Team, Wellcome Sanger Institute Scientific Operations: Sequencing Operations, Wellcome Sanger Institute Tree of Life Core Informatics Team, Tree of Life Core Informatics Collective, Darwin Tree of Life Consortium (2024). The Genome Sequence of the Black Medic, *Medicago lupulina* L.. Wellcome Open Res..

[46] Choi I., Wojciechowski M.F., Steele K.P., Hunter S.G., Ruhlman T.A., Jansen R.K. (2022). Born in the Mitochondrion and Raised in the Nucleus: Evolution of a Novel Tandem Repeat Family in *Medicago polymorpha* (Fabaceae). Plant J..

[47] Ou T., Wu Z., Liu Q., Tian C., Yang Y., Liu L., Guo M., Li Z. (2025). Complete Mitochondrial Genome of *Medicago sativa* ssp. *falcata* (Papilionoideae, Fabaceae): Characterization and Phylogenetic Analysis. Planta.

[48] Christenhusz M.J.M., Fay M.F., Leitch I.J., Royal Botanic Gardens Kew Genome Acquisition Lab, Plant Genome Sizing Collective, Darwin Tree of Life Barcoding Collective, Wellcome Sanger Institute Tree of Life Management, Samples and Laboratory team, Wellcome Sanger Institute Scientific Operations: Sequencing Operations, Wellcome Sanger Institute Tree of Life Core Informatics Team, Tree of Life Core Informatics Collective (2024). The Genome Sequence of Spotted Medick, *Medicago arabica* (L.) Huds. (Fabaceae). Wellcome Open Res..

[49] Tian Y., Wu Z., Tian C., Yang Y., Li Z. (2025). Phylogenetic Classification and Genetic Insights from the Complete Mitochondrial Genome of *Medicago ruthenica*. Front. Plant Sci..

[50] Liu H., Yu J., Yu X., Zhang D., Chang H., Li W., Song H., Cui Z., Wang P., Luo Y. (2021). Structural Variation of Mitochondrial Genomes Sheds Light on Evolutionary History of Soybeans. Plant J..

[51] Asaf S., Khan A.L., Al-Harrasi A., Kim T.H., Lee I.-J. (2018). The First Complete Mitochondrial Genome of Wild Soybean (*Glycine soja*). Mitochondrial DNA B Resour..

[52] Shatskaya N.V., Bogdanova V.S., Kosterin O.E., Vasiliev G.V., Kimeklis A.K., Andronov E.E., Provorov N.A. (2020). The Plastid and Mitochondrial Genomes of *Vavilovia formosa* (Stev.) Fed. and the Phylogeny of Related Legume Genera. Vavilovskii Zhurnal Genet. Selektsii.

[53] Kazakoff S.H., Imelfort M., Edwards D., Koehorst J., Biswas B., Batley J., Scott P.T., Gresshoff P.M. (2012). Capturing the Biofuel Wellhead and Powerhouse: The Chloroplast and Mitochondrial Genomes of the Leguminous Feedstock Tree *Pongamia pinnata*. PLoS ONE.

[54] He Y., Liu W., Wang J. (2023). Assembly and Comparative Analysis of the Complete Mitochondrial Genome of *Trigonella foenum-graecum* L.. BMC Genom..

[55] Katoh K. (2002). MAFFT: A Novel Method for Rapid Multiple Sequence Alignment Based on Fast Fourier Transform. Nucleic Acids Res..

[56] Trifinopoulos J., Nguyen L.-T., von Haeseler A., Minh B.Q. (2016). W-IQ-TREE: A Fast Online Phylogenetic Tool for Maximum Likelihood Analysis. Nucleic Acids Res..

[57] Huson D.H., Scornavacca C. (2012). Dendroscope 3: An Interactive Tool for Rooted Phylogenetic Trees and Networks. Syst. Biol..

